# Evaluation of Pharyngeal Function between No Bolus and Bolus Propofol Induced Sedation for Advanced Upper Endoscopy

**DOI:** 10.1155/2014/248097

**Published:** 2014-03-03

**Authors:** Shinsuke Kiriyama, Hiroshi Naitoh, Minoru Fukuchi, Takaharu Fukasawa, Kana Saito, Yuichi Tabe, Hayato Yamauchi, Tomonori Yoshida, Hiroyuki Kuwano

**Affiliations:** ^1^Department of Surgery, Gunma Chuo General Hospital, 1-7-13 Koun-cho, Maebashi, Gunma 371-0025, Japan; ^2^Department of General Surgical Science, Graduate School of Medicine, Gunma University, 3-39-22, Showa-machi, Maebashi, Gunma 371-8511, Japan

## Abstract

This study aimed to assess pharyngeal function between no bolus and bolus propofol induced sedation during gastric endoscopic submucosal dissection. A retrospective study was conducted involving consecutive gastric cancer patients. Patients in the no bolus group received a 3 mg/kg/h maintenance dose of propofol after the initiation of sedation without bolus injection. All patients in the bolus group received the same maintenance dose of propofol with bolus 0.5 mg/kg propofol injection. Pharyngeal functions were evaluated endoscopically for the first 5 min following the initial administration of propofol. Fourteen patients received no bolus propofol induction and 13 received bolus propofol induction. Motionless vocal cords were observed in 2 patients (14%) in the no bolus group and 3 (23%) in the bolus group. Trachea cartilage was not observed in the no bolus group but was apparent in 6 patients (46%) in the bolus group (*P* < 0.01). Scope stimulated pharyngeal reflex was observed in 11 patients (79%) in the no bolus group and in 3 (23%) in the bolus group (*P* < 0.01). Propofol induced sedation without bolus administration preserves pharyngeal function and may constitute a safer sedation method than with bolus.

## 1. Introduction

Early detection and diagnosis may improve outcome and survival in patients with gastric cancer. The endoscopic submucosal dissection (ESD) technique was developed to resect large neoplasms *en bloc* and to reduce the risk of recurrence [1]. However, ESD generally takes longer than conventional endoscopic mucosal resection (EMR) [2], so adequate moderate and deep sedations are necessary [3].

Recently, propofol sedation administered by anaesthesiologists or gastroenterologists has gained popularity in endoscopic procedures [4–7]. Propofol is a short-acting sedative, that is, an agonist of the **γ**-aminobutyricacid receptor in the central nervous system [8]. Effectiveness of propofol induced sedation during endoscopic procedures is under evaluation. It has been reported that monitored propofol sedation is safer than sedation with midazolam [3, 9, 10]; however, cases of respiratory depression including reduced oxygen saturation during propofol sedation have been reported [11–13]. Bolus administration of propofol is often performed at the initial stage of sedation; however, bolus induction might be regarded as one of the causes of respiratory depression. Although the relationship between propofol and respiratory depression has been reported [14], the direct association with propofol and pharyngeal function is thus far not known. Therefore, we evaluated this association in our study regarding pharyngeal function.

The purpose of this study was to assess differences in pharyngeal function between no bolus propofol induction (NBPI) sedation and bolus propofol induction (BPI) sedation for gastric ESD. The clinical outcomes of gastric ESDs under both NBPI and BPI sedation were retrospectively evaluated and compared with the endoscopic pharyngeal findings, which were originally collected prospectively.

## 2. Methods

### 2.1. Study Design

We analyzed the available patient data of those diagnosed with early gastric cancer and treated endoscopically from March 2010 to January 2011. Patients were excluded if they were <18 years of age; were pregnant; had a history of allergy to sulphite, egg, soybean, or propofol; or did not provide informed consent. Patients with an American Society of Anaesthesiologists (ASA) physical status classification of >2, a severe liver disorder (liver transaminase > 100 IU/L; liver cirrhosis, due to high alcohol intake), severe renal failure (serum creatinine level > 2 mg/dL), severe heart disease (New York Heart Association Class III or IV), mental incompetence, systolic blood pressure < 80 mmHg, and baseline oxygen saturation measured by pulse oximetry of <90% in room air or <95% with oxygen at 2 L/min administered by nasal cannula were also excluded. Informed consent was obtained as per institutional protocol from all patients who underwent endoscopic treatment. This study was performed in accordance with the 1989 revised Helsinki Declaration.

### 2.2. Medication

Local pharyngeal anaesthesia was performed using an 8% topical lidocaine spray prior to intravenous administration of the sedation drugs. For cases before June 2010, ESDs were performed under BPI sedation; for all cases after July 2010, ESDs were performed under NBPI sedation. All patients in the NBPI group received a 3 mg/kg/h maintenance dose of 1% propofol emulsion (10 mg/mL; Astrazeneca, Osaka, Japan) from the initiation of sedation, without bolus administration. Conversely, all patients in the BPI group received a bolus of 0.5 mg/kg of propofol as a loading dose and a 3 mg/kg/h maintenance dose of propofol thereafter, from the initiation of sedation.

Propofol was administered until the patient fell asleep, as determined by a Ramsay sedation score of 5-6. After infusion, a waiting period of approximately 5 min was observed to allow the drug to take complete effect. An automatic infusion pump (Terufusion syringe pump TE-332S; Terumo Corp, Tokyo, Japan) was used to maintain continuous infusion. The objective was to maintain a patient's sedation level between moderate (patient responds properly to verbal commands either alone or accompanied by light tactile stimulation) and deep (patient cannot be easily aroused, but may respond properly to repeated or painful stimulation) [15].

For patients with a body mass index > 22, sedation doses were calculated based on body weight for a body mass index of 22. When a patient seemed to be in discomfort or exhibited restlessness following verbal stimulation during ESD, an additional 10 mg of propofol was given and the infusion rate was increased by 1 mg/kg/h. Conversely, if an adverse event such as hypotension with systolic blood pressure < 80 mmHg or oxygen desaturation < 90% occurred, the maintenance dose was reduced by 1 mg/kg/h. Propofol infusion was continued until removal of the endoscope. All patients received 15 mg of pentazocine at the start of the ESD, as an analgesic agent. The depth of sedation was monitored by a physician not directly involved in the procedure, using a 4-point somnolence score (4, fully alert; 3, awake but lethargic; 2, spontaneous eye closure, but responsive to voice; 1, responds only to shaking or prodding). Endoscopic intubation was initiated once the patient reached a sedation level of 2. All medications were administered by physicians from the endoscopy division who did not participate in the actual endoscopic procedures. Resuscitation equipment was always present in the endoscopic room.

### 2.3. Monitoring

Patients received supplemental oxygen (2 L/min) by nasal cannula in the endoscopic room, and their vital signs and oxygen saturation were continuously monitored and recorded every 5 min using standard three-lead electrocardiogram, pulse oximetry, and automated blood pressure equipment. Chest excursion and respiratory rates were monitored visually and consciousness levels were assessed immediately after the induction of sedation, and then at 20 min intervals thereafter throughout the procedure using the Ramsay sedation score. Vital signs (blood pressure, oxygen saturation, and heart rate) were recorded at the conclusion of the ESD and then at 15, 30, 60, and 120 min after ESD. Patients were discharged from the endoscopy room 15 min after the procedure, provided their vital signs were stable.

### 2.4. Pharyngeal Function

**Figure 1:**
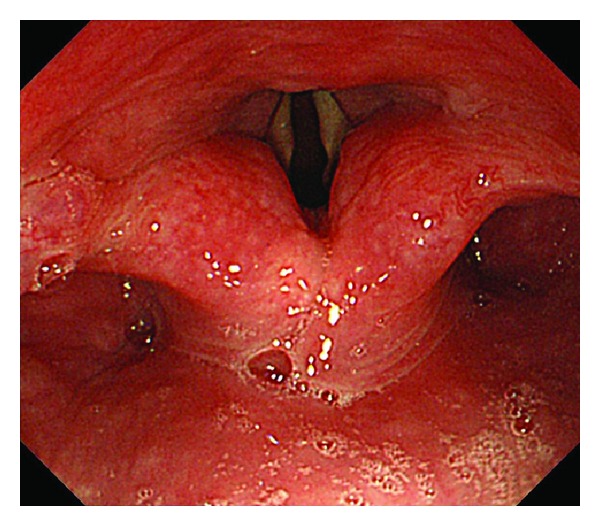
Endoscopic pharyngeal image: no bolus propofol induction patient. Vocal cords: motion; trachea cartilage: no observation.

**Figure 2:**
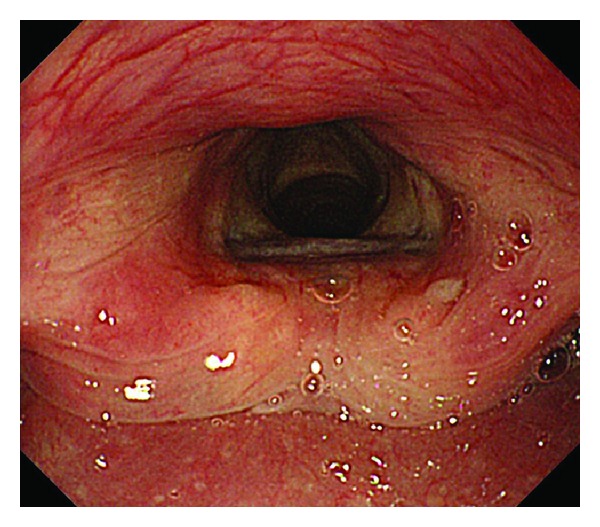
Endoscopic pharyngeal image: bolus propofol induction patient. Vocal cords: motionless; trachea cartilage: observation.

In addition to checking vital signs,pharyngeal functions involving the vocal cords and tracheal cartilage were evaluated endoscopically in the first 5 min after starting propofol infusion. With regard to the vocal cords, “motionless” was defined as endoscopic findings of no movement by continuous opening of the vocal cords of at least 15 seconds to breathe four or five times, and motion was defined as both movement of opening and closure of the vocal cords. Observed tracheal cartilage was defined as trachea cartilage observation by the widely continuous dilatation of vocal cords (Figures 1 and 2). Furthermore, we recorded either existence or nonexistence of pharyngeal reflection based on direct contact with the scope.

### 2.5. Statistical Analysis

Category outcomes were analysed using Fisher's exact test or the chi-square test where appropriate. Continuous outcomes were analysed with the nonparametric Wilcoxon rank sum test for nonnormally distributed data. Continuous variables are expressed as means and standard deviations. Statistical analysis was conducted with SPSS V (Chicago, IL). Statistical significance was defined as *P* < 0.05.

## 3. Results

Twenty-seven early gastric cancer patients were enrolled in this study. Fourteen received no bolus induction of propofol and 13 received bolus induction in the initial stage of sedation during ESDs. The mean age of the NBPI group was 74 years, mean tumour size was 17.2 mm, resection size was 38 mm, and procedure time was 40.4 minutes. In the BPI group, mean age was 69 years, tumour size was 23.6 mm, resection size was 46 mm, and procedure time was 50.9 min. An *en bloc* resection was achieved in all 27 cases. The mean doses of total propofol administered were 227 ± 118 mg in the NBPI group and 228 ± 76 mg in the BPI group. There was no statistically significant difference between the groups in terms of age, gender, tumour size, resection size, procedure time, or mean dose of total propofol (Table 1).

There were no instances of uncontrolled agitation or movement that required a delay during any of the ESD procedures, and none of the patients had to be restrained while under sedation. There were no cases of desaturation to <90% during or after any ESD, and no cases required intubation or ventilation. Similarly, there were no cases of transient hypotension (systolic blood pressure <80 mmHg).

Motionless vocal cords were evident in 2 patients (14%) in the NBPI group and 3 (23%) in the BPI group, but this difference was not significant (Table 2).

Trachea cartilage observation due to continuous wide dilatation of the vocal cords was not seen in the NBPI group (0%) but was apparent in 6 patients (46%) in the BPI group (*P* < 0.01). Scope-stimulated pharyngeal reflex was observed in 11 patients (79%) in the NBPI group and in 3 (23%) in the BPI group (*P* < 0.01).

## 4. Discussion

To our knowledge, this study is the first evaluation of pharyngeal function to compare no bolus-propofol-induced sedation with bolus-propofol-induced sedation administered by gastroenterologists during ESDs for early gastric cancer. Propofol induced sedation without bolus was associated with a higher incidence of the preservation of pharyngeal function during sedation for ESD.

Propofol is a short-acting sedative, with a plasma half-life of only 1–4 min, and the onset of sedation after propofol injection occurs between 30 and 60 s. Stable sedation management is possible via continuous propofol infusion without bolus infusion. Bolus infusion may result in respiratory depression via a sudden increase in the blood concentration of propofol. However, many studies have reported using bolus propofol induced sedation during endoscopy. We therefore compared no bolus induction methods with bolus induction methods for sedation for ESD.

With no reversal agent available for propofol, the presence of personnel that are well trained in airway rescue is mandatory, although none of the patients in either group in this study required endotracheal intubation. Furthermore, there were no complications in neither the BPI nor the NBPI method. However, there is the risk of salivary aspiration during ESD because most of ESDs are treatment for the long time more than 60 minutes. Thus, to reduce the aspiration risk, it is desirable for the pharyngeal function to be maintained during the procedure and sedation. Therefore, it is thought that propofol-induced sedation without bolus can negate respiratory depression at the initial stage of sedation.

In the NBPI group, there was a lower rate of trachea cartilage observation, and a higher rate of positive pharyngeal reflex by scope stimulation, than in the BPI group. There was no significant difference in movement of the vocal cords between the groups. Low trachea cartilage observation and high pharyngeal reflex by scope stimulation in the NBPI group suggested that pharyngeal functions were preserved to a greater extent in this group than in the BPI group. In fact, propofol sedation without bolus administration is safer than with initial bolus administration.

Our study demonstrates that the induction and maintenance of sedation can be safely performed using propofol, thus avoiding respiratory dysfunction. The Ramsay score of 5-6 that we obtained in most cases also indicates that the doses of propofol that we calculated were appropriate for achieving moderate to deep sedation. In addition, the rapid onset and offset of sedation associated with the continuous infusion of propofol decreases the risk of sedation complications and reduces the burden on the gastroenterologist during the procedure.

Our goal was to maintain a moderate to deep sedation level. Given the narrow therapeutic window of propofol, fluctuations in the depth of sedation may occur, but none of the ESD procedures in this study had to be delayed or terminated, suggesting that our dose calculations and sedation procedures were effective, as well as safe.

The limitation of this study is to be a study performed in small sample size and only single hospital. No severe adverse events, such as desaturation or hypotension, resulted from the administration of propofol by a nonanaesthesiologist physician in this sample size. However, additional training has been recommended to be provided for the safe administration of propofol [16, 17]. An ASA/ASGE taskforce further recommended that nonanaesthesiologists using propofol for endoscopic procedures should be trained by anaesthesiologists [18]. Propofol has a narrow therapeutic window that can result in a rapid depression of consciousness and cardiovascular function, leading to a state of general anaesthesia, and there is no reversal agent. Therefore, additional training and the use of a safe infusion protocol are recommended to ensure the safe administration of propofol. As the second limitation of our study, the endoscopists were not blinded. To the best of our knowledge, there is no evidence in the literature about association between the endoscopic pharyngeal findings and respiratory depression during sedation of endoscopic procedures. Hence, further evaluation about the association between the endoscopic pharyngeal findings and respiratory depression during sedation might be necessary.

In conclusion, vocal cords function and pharyngeal reflex in gastric ESD patients received propofol induced sedation without bolus administration at initial stage were significantly higher than in propofol induced sedation with bolus administration. As propofol induced sedation without bolus preserves pharyngeal function and can negate respiratory depression at the initial stage of sedation, it represents a safe, useful sedation method.

## Figures and Tables

**Table 1:** Patient characteristics.

	NBPI	BPI	*P* value
Number of cases	14	13	
Mean age (years) (range)	74 (56–84)	69 (59–83)	NS
Gender (male/female)	9/5	7/6	NS
Tumor size (mm)	17.2 ± 6.6	23.6 ± 9.7	NS
Resection size (mm)	38.0 ± 14.0	45.5 ± 10.1	NS
Procedure time (min)	40.4 ± 20.4	50.9 ± 19.8	NS
Mean total dose of propofol (mg)	227 ± 118	228 ± 76	NS
Complications	0	0	NS

NBPI: no bolus propofol induction; BPI: bolus propofol induction; NS: not significant.

**Table 2:** Comparison of respiratory function.

	NBPI	BPI	*P* value
Vocal cords			NS
Motion	12 (86%)	10 (77%)	
Motionless	2 (14%)	3 (23%)	
Trachea cartilage			0.0058
No observation	14 (100%)	7 (54%)	
Observation	0 (0%)	6 (46%)	
Scope stimulated pharyngeal reflex			0.007
Positive	11 (79%)	3 (23%)	
Negative	3 (21%)	10 (77%)	
